# Saddle Nose with Recurrent Sinusitis and Arthralgias: a Not-to-lose Diagnosis

**DOI:** 10.31138/mjr.33.1.91

**Published:** 2022-03-31

**Authors:** Vassiliki Syrmou, Theodora Simopoulou, Dimitrios P. Bogdanos, Ioannis Alexiou

**Affiliations:** Department of Rheumatology and Clinical Immunology, Faculty of Medicine, University of Thessaly, General University Hospital of Larissa, Larissa, Greece

**Figure 1.**
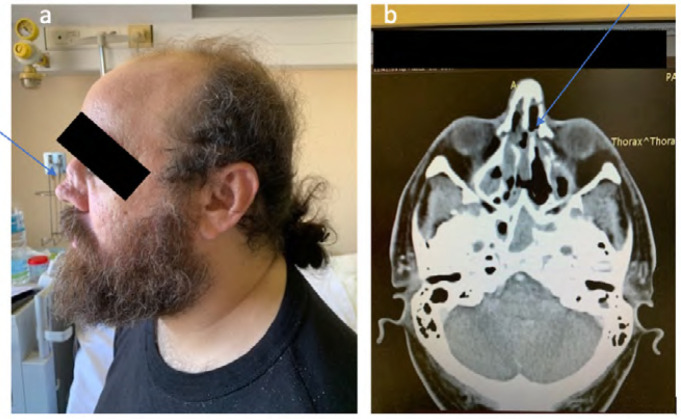
**(A)** saddle nose on profile; **(B)** nasal septum perforation with hyperplasia of the nasal mucosa.

**Figure 2.**
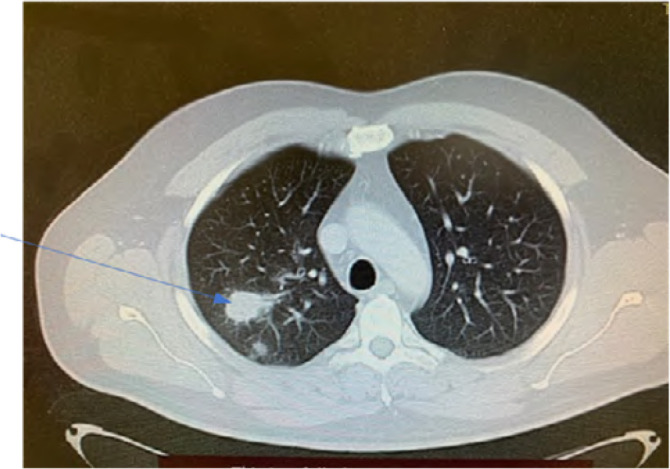
CT thorax with pulmonary granulomas.

Nasal and lung features in a patient with Granulomatosis with Polyangiitis (GPA, formerly called Wegener’s). A 39-year-old man presented with a 6-month history of recurrent sinusitis, nose deformity (**Figure 1A**), low grade fever and arthralgias. ENT examination revealed nasal septum perforation (**Figure 1B**), and sensorineural hearing loss. He complained about dry cough and CT thorax (**Figure 2**) revealed multiple granulomas bilaterally. Nasal mucosa biopsy revealed signs of acute and subacute inflammatory changes with infiltration of neutrophils and histiocytes. From urine microscopy there was microscopic haematuria with glomerular red blood cells. Patient had high c-ANCA (1/160) and PR3(>100 RU/ml, NV<20 RU/ml) titre. After setting the diagnosis of polyarteritis with granulomatosis (PGA), symptoms regressed with methylprednisolone pulses and cyclophosphamide. Despite immunosuppression, nasal septum deformity requires surgical repair.

